# The Impact of integrated management of childhood illness training on knowledge levels of healthcare workers in Botswana

**DOI:** 10.1371/journal.pgph.0003899

**Published:** 2025-02-24

**Authors:** Kebayaone P. Gare, Keatlaretse Siamisang, Keemenao Ramogalana, Omphemetse Mafala, Orontshamang Salepito, Marinah Seobakeng, Lesego Kuate-Lere, John T. Tlhakanelo

**Affiliations:** 1 Department of Family Medicine & Public Health, Faculty of Medicine, University of Botswana, Gaborone, Botswana; 2 Department of Public Health, Ministry of Health, Gaborone, Botswana; PLOS: Public Library of Science, UNITED STATES OF AMERICA

## Abstract

The Integrated Management of Childhood Illness (IMCI) strategy was developed to improve outcomes through integration of preventive and curative interventions in countries with high mortality. This study aimed to assess the impact of IMCI training on the knowledge levels of healthcare workers (HCWs) in Botswana, comparing the trained with the non-trained. This was a cross-sectional study from a national IMCI survey across all 27-health districts of Botswana in September 2023. Within each district, random sampling was used to select 10 healthcare facilities (HCFs) to be included in the survey. HCFs were ordered by their size to ensure that all types were represented. The demographics, training and qualifications of the HCWs were documented. Stata 13.1 software was used for analysis, and data was summarized with frequencies and percentages. Pearson’s chi square test was used to compare the performances of IMCI trained versus non-IMCI trained HCWs. A p-value of <0.05 was considered statistically significant. A total of 964 HCWs participated in the survey. The most frequent cadre was General Nurse with 385 (40.7%) participants. Participants trained in IMCI were 471 (49.2%) and those who consult sick children were 615 (65.1%). Those who were IMCI trained had good (80–100%) and moderate (60–79%) knowledge levels at 51 (14.1%) and 91 (25.2%) participants respectively than those who had no IMCI training at 25 (9.9%) for good and 52 (20.6%) for moderate performances. HCWs who were not IMCI trained had poor performance (0-59%) at 175 (69.4%) participants while the IMCI trained had poor performance at 219 (60.7%). The performances showed no statistically significant difference (p = 0.092), reflective of similar knowledge levels. Overall, the performance of IMCI trained HCWs was not significantly different from those not IMCI trained, translating to that IMCI training does not have effect on knowledge levels of HCWs in management of childhood illness. This, however, should be interpreted with caution since it could be due to the stated study limitations. A future IMCI study on knowledge, attitudes and practices (KAP) or a longitudinal study would be more beneficial.

## Introduction

Under-five mortality has been a major public health challenge from time immemorial [1]. More than 7.5 million children younger than age five living in low- and middle-income countries (LMIC) die every year [2]. The introduction of the World Health Organization (WHO)’s IMCI guidelines in the mid-1990s contributed to global reductions in under-five mortality rate (U5MR) [3]. The WHO developed the IMCI strategy to reduce mortality and morbidity and to improve quality of care by advancing the delivery of a variety of curative and preventive medical and behavioral interventions at HCFs, in homes, and in the community [2]. In 1995, guidelines for the IMCI at first-level facilities were finalized in a collaborative effort led by the WHO [4]. The United Nations Children’s Fund (UNICEF) and WHO promoted a training course based on these guidelines in 1996, which targeted HCWs in first-level facilities [5].

For many countries, the introduction of IMCI training provides an opportunity to review child health policies and to reorganize services and interventions [6]. IMCI training offers a holistic training package focusing on the main causes of under-five mortality [7]. It focuses on improving case management among HCWs, health system strengthening and promotion of good practices at both the family and community levels [8]. One of the learning outcomes of the training is the acquisition of technical or motor skills by HCWs [7]. A 2016 Cochrane review found that IMCI was associated with a 15% reduction in child mortality when activities were implemented in HCFs and communities [2,7]. IMCI has been associated with a reduction of 13% of under-five mortality in Tanzania and a doubling in the annual rate of reduction of under-five mortality in Egypt (3.3% vs. 6.3%) [9,10]. IMCI is part of the national child strategy in 90 of the 97 LMIC and is the primary strategy for child health according to the WHO [11].

Botswana introduced the IMCI strategy in 1998 [1]. Since then, IMCI training has been done routinely at the end of nurses’ training and for the district level as needed. Reductions in the U5MR have been documented, although these are not on par with the expected Sustainable Development Goal (SDG) 3 projections [1]. A child mortality audit in Botswana between 2011 and 2013 demonstrated that 46% of paediatric in-hospital deaths were due to severe pneumonia, diarrhoeal dehydration and sepsis. Thirty-three percent (33%) of in-hospital paediatric deaths occurred within the first 24 hours, an indication that children arrived critically ill [12]. Botswana’s health services are mostly public with a growing private sector [13]. The healthcare system is based on a primary healthcare model and services are provided through a network of HCFs [13]. Access to a HCF does not always translate to good health service, as many of the facilities are severely short staffed [13]. In HCFs, the IMCI strategy promotes the accurate identification of childhood illnesses in outpatient settings, ensures appropriate combined treatment of all major conditions that affect a young child, strengthens the counselling of caretakers, and speeds up the referral of severely ill newborns and children [11]. For this reason, there is extreme need for training of HCWs in IMCI in Botswana. Scaling up standard IMCI in Botswana (as in many other developing countries) has faced a number of challenges [14–17]. The standard face-to-face training for the IMCI continues to be plagued by concerns of low coverage of trainees, the prolonged absence of trainees from the HCFs to attend training and the high cost of training [8]. The comprehensive implementation of the IMCI strategy has the potential to significantly influence the U5MR in Botswana [1]. In the area of health, training evaluation can be done through a systematic collection of data to evaluate the performance of HCWs in assessing and treating patients [7]. From literature review there hasn’t been any local IMCI studies looking at the KAP of HCWs in Botswana. There was only one published study at the time, which was about “Implementation of the IMCI strategy: challenges and recommendations in Botswana” [1]. The mentioned study only focused on one health district. Along with the implementation of the 2023 IMCI national survey, our study would bridge this research gap. This study aimed to assess the impact of IMCI training on the knowledge levels of HCWs across all health districts in Botswana, comparing the IMCI trained versus non-IMCI trained from the 2023 national survey data.

## Methods

### Study design

The study was part of a HCF survey using a standard audit approach. This was a cross-sectional study assessing the effect of IMCI training by focusing on how HCWs manage cases of children presenting to outpatient HCFs with common childhood illnesses including fever, diarrhea, cough or difficulty breathing or ear problems.

### Study sites and approach

The survey was conducted across all 27-health districts of Botswana. Within the districts, a random sampling technique was used to select HCFs to be included in the survey. Ten (10) HCFs were selected within each health district. Selections were made from all HCF levels of hospitals, clinics with maternity, other clinics and health posts. The survey exercise was conducted over a day in each facility. Selection of 10 HCFs per district was done mainly to balance the need to cover as many facilities in each district as possible (to enhance generalizability) and the limited resources (including human resources, finances and time).

### Sampling and sampling frame

A list of all HCFs in the districts was used as a sampling frame for the survey. HCFs were ordered by their size to ensure all types of HCFs (health posts, clinics without maternity, clinics with maternity and hospitals) were represented. HCFs were excluded if: they did not provide sick child services; they were not functional; they were not open regularly during the week; they were too isolated to be reached during the survey period; or they saw too few sick children daily (less than 4 sick children/day). The decision to exclude those facilities was also done in collaboration with district health management teams. The investigators approached all eligible HCWs present on the day of the interview. Consenting HCWs were enrolled in the study, i.e., the sampling was exhaustive.

### Data collection

Before data collection, there was a week-long formal training of supervisors and surveyors. The training was on interpretation and administration of the survey forms and general survey procedures. Piloting was done before commencement of data collection and adjustments were made to the questionnaire following pilot results, for clear comprehension by participants. The data collection tool was derived from pages 100 to 118 of the WHO health facility guide [18]. It is a tool to evaluate the quality of care delivered to sick children attending outpatient facilities [18]. The tool has been validated in African populations and used for the two previous IMCI surveys that were conducted in Botswana. The health facility guide represents a resource package for conducting standardized health facility surveys [19]. Recruitment of participants was done from 11^th^ to 22^nd^ September 2023 by different teams across the country. The demographics, training and qualifications of the HCWs were documented.

### Data analysis

Data was entered into Microsoft excel. After data cleaning and preparation, Stata 13.1 software was used for analysis. Categorical data was summarized with frequencies and percentages while numerical data was summarized with medians (& interquartile ranges). Pearson’s chi square test was used to compare different categorical data. Chi square test was chosen because the data was mainly categorical in nature. It was the suitable test to compare knowledge levels between IMCI trained versus non-IMCI trained HCWs. The HCWs’ performance on the questions on diagnosis and assessment for common childhood illnesses was summarised into a table (available as S3 File). The total knowledge scores of each individual were computed through Stata 13.1 by adding their individual scores for each question, to make the maximum knowledge score 21. The knowledge of individual HCWs were then categorised according to Bloom’s cutoffs, where 80–100% represents good performance, 60–79% represents moderate performance, and <60% represents poor performance. A p-value of <0.05 was considered to demonstrate significant differences between variables.

### Ethical considerations

Ethical clearance was obtained from Ministry of Health (MoH) Ethics Committee and the approval number was Reference No.: HPRD: 6/14/1. Participants were not subjected to any procedures or activities that may harm or cause them any degree of discomfort and they provided written informed consent before participation. Participants’ data is presented in a way that would not compromise their privacy or confidentiality. All identifiers were removed and coding was used so that participants cannot be identified.

## Results

A total of 964 HCWs participated in the 2023 National IMCI survey. Table 1 shows the characteristics of HCWs who were interviewed in this survey. Female HCWs comprised 665 (69.1%) of all the HCWs partaking in the survey meaning that they were the majority. Most of these HCWs, 667 (69.2%), were based in clinics while others were based in hospitals, 27 (2.8%) and health posts, 270 (28.0%). The most frequent cadre was General Nurse, 385 (40.7%) followed by Health Education Officer, 160 (16.9%) and Midwife, 158 (16.7%). Those who were employed for more than 20 years were 259 (27.4%) followed by those who worked for 0-4 years at 210 (22.2%) participants. Almost half, 471 (49.2%) indicated that they underwent IMCI training while 486 (50.8%) did not. Diploma holders, 592 (65.3%) constituted the majority of the participants followed by certificate or lower then degree or higher. Six hundred and fifteen (65.1%) indicated that they regularly consult sick children.

**Table 1:** Characteristics of participants (whole cohort) n = 964.

Characteristic	Number (%)
**Sex**	
*Data available*	*962 (99.8)*
Female	665 (69.1)
Male	297 (30.9)
**Age (years)**	
*Data available*	*926 (96.1)*
*Median (IQR)*	37 (30-46)
<30	196 (21.2)
30–39	336 (36.3)
40–49	237 (25.6)
50–59	133 (14.4)
≥ 60	24 (2.6)
**Facility Type**	
*Data available*	*964 (100)*
Hospital	27 (2.8)
Clinic	667 (69.2)
Health Post	270 (28.0)
**Work (Cadre)**	
*Data available*	*947 (98.2)*
General Nurse	385 (40.7)
Midwife	158 (16.7)
Family Nurse Practitioner	40 (4.2)
Other Post Basic Nurse	21 (2.2)
General Doctor	26 (2.7)
Health Care Auxiliary (HCA)	50 (5.3)
Health Education Officer (HEO)	160 (16.9)
Pharmacy Technician	79 (8.3)
Other	28 (3.0)
**Duration of Employment (years)**	
*Data available*	*946 (98.1)*
0–4	210 (22.2)
5–9	191 (20.2)
10–14	152 (16.1)
15–19	134 (14.2)
≥ 20	259 (27.4)
**IMCI Training**	
*Data available*	*957 (99.3)*
Yes	471 (49.2)
No	486 (50.8)
**Highest Qualification**	
*Data available*	*907 (94.1)*
Degree or Higher	118 (13.0)
Diploma	592 (65.3)
Certificate or Lower	196 (21.6)
**Duration (years) of work at Facility**	
*Data available*	*921 (95.5)*
0–4	679 (73.7)
5–9	161 (17.5)
10–14	34 (3.7)
15–19	13 (1.4)
≥ 20	34 (3.7)
**Consultation of sick children**	
*Data available*	*945 (98.0)*
Yes	615 (65.1)
No	330 (35.0)

Table 2 displays the characteristics of HCWs stratified by IMCI training. Regarding the distribution by sex, 471 (48.9%) of the HCWs were IMCI trained while 486 (50.4%) were not IMCI trained. For age, there was a statistically significant difference (p < 0.001) in the frequencies between those IMCI trained and not IMCI trained. There was no statistically significant difference (p = 0.638) in the frequencies between the IMCI trained and not IMCI trained based on the type of HCF. Work (cadre), duration of employment, and highest qualification had statistically significant differences (p < 0.05) between those IMCI trained and those not IMICI trained. In terms of those HCWs who regularly consult sick children, IMCI trained versus not IMCI trained had a statistically significant difference (p < 0.001).

**Table 2:** Characteristics of participants (stratified by IMCI training).

Variable	IMCI trained (%) n = 471	Not IMCI trained (%) n = 486	IMCI training unknown (%) n = 7	Totals (%) n = 964	p-value
**Sex**					
*Data available*	*471 (100.0)*	*484 (99.6)*	7(100.0)	*962 (99.8)*	0.059
Female	311 (66.0)	347 (71.7)	7(100.0)	665 (69.1)	
Male	160 (34.0)	137 (28.3)	0(0.0)	297 (30.9)	
**Age (years)**					
*Data available*	*447 (94.9)*	*473 (97.3)*	6(85.7)	*926 (96.1)*	<0.001*
<30	72 (16.1)	123 (26.0)	1(14.3)	196 (20.3)	
30–39	184 (41.2)	149 (31.5)	3(42.9)	336 (34.9)	
40–49	110 (23.4)	125(26.4)	2(28.6)	237 (24.6)	
50–59	73 (15.5)	60 (12.7)	0(0.0)	133 (13.8)	
≥ 60	8 (1.7)	16 (3.4)	0(0.0)	24 (2.5)	
**Facility Type**					
*Data available*	*471 (100)*	*486 (100)*	7(100)	*964 (100)*	0.638
Hospital	13 (2.8)	14 (2.9)	0(0.0)	27 (2.8)	
Clinic	333 (70.7)	330 (67.9)	4(57.1)	667 (69.2)	
Health Post	125 (26.5)	142 (29.2)	3(42.9)	270 (28.0)	
**Work (Cadre)**					
*Data available*	*463 (98.3)*	*479(98.6)*	5(71.4)	*962 (99.8)*	<0.001*
General Nurse	232 (50.1)	152 (31.7)	1(14.3)	385 (39.9)	
Midwife	87 (18.8)	70 (14.6)	1(14.3)	158 (16.4)	
FNP	25 (5.4)	15 (3.1)	0(0.0)	40 (4.3)	
Other Post Basic N.	10 (2.2)	11 (2.3)	0(0.0)	21 (2.2)	
General Doctor	8 (1.7)	18 (3.8)	0(0.0)	26 (2.8)	
HCA	6 (1.3)	44 (9.2)	0(0.0)	50 (5.3)	
HEO	91 (19.7)	69 (14.4)	0(0.0)	160 (17.0)	
Pharm. Tech	4 (0.9)	73 (15.2)	2(28.6)	79 (8.2)	
Other	0 (0.0)	27 (5.6)	1(14.3)	28 (2.9)	
**Duration of Empl. (years)**					
*Data available*	*465 (98.7)*	*475 (97.7)*	6(85.7)	*946(98.1)*	<0.001*
0–4	70 (15.1)	138 (29.1)	2(28.6)	210 (21.8)	
5–9	113 (24.3)	77 (16.2)	1(14.3)	191 (19.8)	
10–14	85 (18.3)	66 (13.9)	1(14.3)	152 (15.8)	
15–19	55 (11.8)	78 (16.4)	1(14.3)	134 (13.9)	
≥ 20	142 (30.5)	116 (24.4)	1(14.3)	259 (26.9)	
**Highest Qualification**					
*Data available*	*447 (94.9)*	*456 (93.8)*	4(51.1)	*907 (94.1)*	0.003*
Degree or Higher	47 (10.5)	71 (15.6)	0(0.0)	118 (12.2)	
Diploma	317 (70.9)	274 (60.1)	2(28.6)	593 (61.5)	
Certificate or Lower	83 (15.6)	111 (24.3)	2(28.6)	196 (20.3)	
**Duration (years) of work at Facility**					
*Data available*	*454 (96.4)*	*461 (94.9)*	6(85.7)	*927 (96.2)*	0.241
0–4	330 (72.7)	343 (74.4)	6(85.7)	679 (70.4)	
5–9	77 (17.0)	84 (18.2)	0(0.0)	161 (16.7)	
10–14	16 (3.5)	18 (3.9)	0(0.0)	34 (3.5)	
15–19	9 (2.0)	4 (0.9)	0(0.0)	13 (1.4)	
≥ 20	22 (4.9)	12 (2.6)	0(0.0)	34 (3.5)	
**Consultation of sick children**					
*Data available*	469 (99.6)	470 (96.7)	6(85.7)	945(98.7)	<0.001*
Yes	361 (77.0)	252 (53.6)	2(28.6)	615 (63.8)	
No	108 (23.0)	218 (46.4)	4(57.1)	330 (34.2)	

* Statistically significant: p-value < 0.05, FNP- Family Nurse Practitioner, N.-Nurse, Health Care Auxiliary, HEO- Health Education Officer, Pharm. Tech- Pharmacy Technician, Empl.- Employment.

Table 3 shows the characteristics of HCWs who consult sick children regularly, stratified by IMCI training. Among them, 400 (65.1%) were female. The most frequent age group was 30-39 years at 234 (39.7%) participants. General Nurses, 377 (63.4%) were the ones who mostly consulted sick children followed by Midwives, 134 (22.6%). Those who had been employed for 20 years or more formed a quarter, 154 (25.4%) and they were the most frequent among the duration of employment category. A third, 112 (33.7%) of the HCWs who participated in this survey were trained in IMCI between 2015- 2019.

**Table 3:** Characteristics of healthcare workers who consult sick children (stratified by IMCI training).

Variable	IMCI trained (%) n = 361	Not IMCI trained (%) n = 252	IMCI training Unknown (%) n = 2	Totals (%) n = 615	p-value
**Sex**					
*Data available*	361 (100.0)	251 (99.6)	2 (100.0)	614 (99.8)	0.411
Female	230 (63.7)	168 (66.9)	2 (100.0)	400 (65.1)	
Male	131 (36.3)	83 (33.1)	0 (0.0)	214 (34.9)	
**Age (years)**					
*Data available*	341 (94.5)	247 (98.0)	2 (100.0)	590 (95.9)	0.029*
<30	70 (20.5)	62 (25.1)	1(50.0)	133 (22.5)	
30–39	154 (45.1)	80 (32.4)	0 (0.0)	234 (39.7)	
40–49	73 (21.4)	64 (25.9)	1 (50.0)	138 (23.4)	
50–59	37 (10.9)	31 (12.6)	0 (0.0)	68 (11.5)	
≥ 60	7 (2.1)	10 (4.0)	0 (0.0)	17 (2.9)	
**Facility Type**					
*Data available*	361 (100.0)	252 (100.0)	2 (100.0)	615 (100)	0.526
Hospital	12 (3.3)	13 (5.2)	0 (0.0)	25 (4.1)	
Clinic	264 (73.1)	180 (71.4)	1 (50.0)	445 (72.4)	
Health Post	85 (23.6)	59 (23.4)	1 (50.0)	145 (23.6)	
**Work (Cadre)**					
*Data available*	346 (95.8)	246 (97.6)	2 (100.0)	594 (96.6)	0.259
General Nurse	228 (65.0)	148 (59.4)	1 (50.0)	377 (63.4)	
Midwife	77 (21.9)	56 (22.5)	1 (50.0)	134 (22.6)	
FNP	23 (6.6)	14 (5.7)	0 (0.0)	37 (6.2)	
Other Post Basic Nr.	10 (2.9)	10 (4.0)	0 (0.0)	20 (3.4)	
General Doctor	8 (2.3)	18 (7.2)	0 (0.0)	26 (4.4)	
**Duration of Employment (years)**					
*Data available*	357 (98.9)	247 (98.0)	2 (100.0)	606 (98.5)	<0.001*
0–4	63 (17.6)	62 (25.1)	1 (50.0)	126 (20.8)	
5–9	91 (25.5)	34 (13.8)	0 (0.0)	125 (20.6)	
10–14	78 (21.8)	37 (15.0)	0 (0.0)	115 (19.0)	
15–19	41 (11.5)	45 (18.2)	0 (0.0)	86 (14.2)	
≥ 20	84 (23.5)	69 (27.9)	1 (50.0)	154 (25.4)	
**Year of Training**					
*Data available*	327 (90.6)	4 (1.6)	1 (100.0)	332 (54.0)	<0.001*
Before 2010	85 (26.0)	0 (0.0)	1 (100.0)	86 (25.9)	
2010–2014	92 (28.1)	1 (25.0)	0 (0.0)	93 (28.0)	
2015–2019	111 (33.9)	1 (25.0)	0 (0.0)	112 (33.7)	
2020 to date	39 (11.9)	2 (50.0)	0 (0.0)	41 (12.3)	
**Highest Qualification**					
*Data available*	331 (91.7)	57 (22.6)	1 (100.0)	389 (63.3)	0.003*
Degree or Higher	41 (12.4)	9 (15.8)	0 (0.0)	50 (12.9)	
Diploma	290 (87.6)	48 (84.2)	1 (100.0)	339 (87.1)	
**Duration (years) of work at Facility**					
*Data available*	330 (91.4)	56 (22.2)	1 (100.0)	387 (62.9)	0.722
0–4	269 (81.5)	45 (80.4)	1 (100.0)	315 (81.4)	
5–9	54 (16.4)	9 (16.1)	0 (0.0)	63 (16.3)	
10–14	7 (2.1)	2 (3.6)	0 (0.0)	9 (2.3)	
15–19	0 (0.0)	0 (0.0)	0 (0.0)	0 (0.0)	
≥ 20	0 (0.0)	0 (0.0)	0 (0.0)	0 (0.0)	

* Statistically significant p-value (<0.05).

Table 4 shows the overall performance of HCWs who consult sick children on knowledge questions, stratified by IMCI training. A total of 615 HCWs regularly consulted sick children. Three hundred and sixty-one (58.7%) of the participants were IMCI trained while 252 (41.0%) were not IMCI trained. Those who were IMCI trained had good (80–100%) and moderate (60–79%) performances at 51 (14.1%) and 91 (25.2%) participants respectively than those who had no IMCI training, i.e., at 25 (9.9%) and 52 (20.6%) respectively. HCWs who were not IMCI trained had poor performance (<60%) at 175 (69.4%) participants while the IMCI trained had poor performance at 219 (60.7%) participants.

**Table 4:** Overall knowledge levels of HCWs’ who consult sick children on the management of common childhood illnesses in outpatient settings (stratified by IMCI training).

Knowledge levels	IMCI trained (%) n = 361	Not IMCI trained (%) n = 252	IMCI training unknown (%) n = 2	Total (%) n = 615
Good (80–100%)	51 (14.1)	25 (9.9)	1 (50.0)	77 (12.5)
Moderate (60–79%)	91 (25.2)	52 (20.6)	0 (0.0)	143 (23.3)
Poor (0–59%)	219 (60.7)	175 (69.4)	1 (50.0)	395 (64.2)
*Total*	*361 (100.0)*	*252 (100.0)*	*2 (100.0)*	*615 (100.0)*

Pearson chi-square = 7.9944, p-value: 0.092.

## Discussion

One of the goal targets for SDG 3 is to end preventable deaths of newborns and children under 5 years of age by 2030, with all countries aiming to reduce neonatal mortality to at least as low as 12 per 1,000 live births and under-5 mortality to at least as low as 25 per 1,000 live births [20]. We set out to assess the impact of IMCI training on the knowledge levels of HCWs towards IMCI in Botswana from national survey data. This was the fourth national IMCI survey conducted following the 2004, 2012 and 2017 surveys since Botswana aims to conduct these surveys every five years. More (964) HCWs participated in this survey compared to the years 2012 and 2017 in which 517 and 243 HCWs participated respectively. This was expected because the 2023 survey covered all the 27 health districts while the previous two did not cover all [21,22]. For the 2017 survey, 58/243 (37%) were IMCI trained compared to 471/957 (49.2%) for the current study. This increase was expected because the survey for the current study covered all districts and most likely because the country trained additional HCWs on IMCI after 2017 despite challenges like the COVID-19 pandemic. For the 2017 survey, 174/243 (72.0%) was those who manage sick children and for the 2023 survey, 615/945 (65.1%) was those who consult sick children [21]. Even though the proportions decreased between the surveys, the absolute numbers increased and this could be attributable to more districts being covered in the latter.

For those who consult sick children regularly, there was no statistically significant difference (p = 0.259) in knowledge levels between IMCI trained cadres versus cadres not trained in IMCI for the current study. It is possibly due to that managing sick children regularly increases provider knowledge as demonstrated by a study by Meaney PA et al. Their study demonstrated that care of the seriously ill child significantly increased provider knowledge and loss of knowledge occurred over time [12,23]. Furthermore, IMCI training did not significantly impact overall knowledge acquisition or retention, while professional status impacted overall score and lost to follow-up impacted knowledge retention [12]. Most participants in the current study, 429 (69.5%) recognised that IMCI guidelines are ‘very useful’ in the clinical management of sick children. This is comparable to a study conducted in 2022 in Oman which aimed to assess the IMCI pre-service training perceptions of medical students, including their willingness to prospectively utilize the IMCI guidelines in clinical settings [24]. The medical knowledge and clinical practice skill enhancement abilities of the IMCI sessions were recognized by ≥49.7% of medical students [24]. The overall results of this study pointed to the clinical practice implications, based on the positive student perceptions, of the IMCI pre-service training [24].

A quantitative study was done to identify the problems IMCI implementers face when tending children under 5 years in the Gaborone Health District of Botswana [1]. The study population was made up of all the IMCI trained and registered nurses, and systematic sampling was used to randomly select study participants [1]. The study findings indicated challenges related to low training coverage, health systems, and the unique features of the IMCI strategy [1]. Some of these findings are in agreement to those of our study because ours shows that less than half (471/964) of HCWs who participated were IMCI trained, confirming almost low training coverage. Similarly, one study aimed to explore the challenges encountered in implementing the IMCI strategy guidelines by primary healthcare (PHC) nurses at first level HCFs in the Francistown area of Botswana [25]. PHC nurses stated that the implementation of the IMCI strategy could be enhanced if the lack of resources, shortage of staff, lack of time, untrained staff and lack of supervision could be addressed effectively [25]. The study concluded that HCWs require sufficient human and material resources, sustained in-service training and effective follow-up supervision to continue to implement the IMCI guidelines effectively at PHC level [25]. Comparing this to the current study, there was an issue of untrained staff, which relates to low training coverage in the current study. From both studies it shows that low training has been an issue that needs to be addressed from way back.

In the Botswana setting HCWs are trained through a shorter (6-Day) IMCI course, which is a 7-day course in some countries. The current study showed no difference (p = 0.092) in the knowledge levels between IMCI trained versus non-IMCI trained HCWs, a finding which was not expected and could be due to the nature of the study data, which was largely categorical. Categorical data can’t be used for complex mathematical operations or to calculate deeper metrics like means or standard deviations. Instead, categorical data is analysed using frequencies, proportions, or percentages. A relatable cross-sectional evaluation in Afghanistan compared knowledge before and after training, and HCW performance in assessment, classification and treatment of sick children in two similar cohorts, eight months post-training [26]. The mean increase in knowledge scores of the thirty 7-Day course trainees was 29 [95% Confidence Interval (CI): 24, 34] compared to 23 (95% CI: 18, 28) in the 31 trained in the 11-Day course [26]. The study concluded that given similar performance and knowledge of HCWs trained in both courses, potential cost savings, the possibility of training more HCWs and the relative ease with which HCWs in remote settings might participate in a shorter course, it seems prudent to standardize the 7-Day course in Afghanistan where child mortality rates remain unacceptably high [26]. One quasi-experimental study was conducted in two provinces of Pakistan in which 104 HCWs were conveniently selected to receive either the abridged (7-day) or the standard (11-day) training [27]. The improvement in mean knowledge scores of the 7-day and 11-day training groups was 31.6 (95% CI 24.3, 38.8) and 29.4 (95% CI 23.9, 34.9) respectively, p = 0.630 while the improvement in mean clinical skills scores of the 7-day and 11-day training groups was 23.8 (95% CI: 19.3, 28.2) and 23.0 (95% CI 18.9, 27.0) respectively, p = 0.784 [27]. The study concluded that abridged IMCI training is equally effective as the standard training. However, training for certain illnesses may be better delivered by the standard course [27]. In contrast to the Botswana setting, the current study showed no statistically significant difference (p = 0.092) in knowledge levels of the IMCI trained as opposed to non-IMCI trained HCWs since the training delivered here is through the abridged (6-day) course as well. In rural Malawi, one related intervention study aimed to assess the impact of 1) portable pulse oximeters and 2) IMCI continued education training on the diagnosis and treatment of non-malarial fever amongst paediatric patients being treated by the Global AIDS Interfaith Alliance (GAIA) [28]. The pre- and post-exam scores for the providers who participated in the IMCI training showed an increase in content knowledge and understanding (p < 0.001) [28]. An increase in pneumonia diagnoses was detected for patients who received pulse oximeter evaluation with oxygen saturation <95% (p < 0.001) [28]. The findings of this study are different from ours, in which, knowledge levels seemed not to change for those who were IMCI trained and those who are not. These findings were not expected since training is expected to increase knowledge levels among HCWs.

### Strengths and limitations

The strength of this study lies in the fact that the survey, from which data was analysed, was the first IMCI national survey that comprehensively included all the health districts in Botswana among the four IMCI surveys conducted. The study however was not without limitations. Some of the furthest HCFs in some districts did not participate in the survey due to unavailability of HCWs (mostly nurses) who were on day offs or had to refer patients to the next level of care. In addition, the research data of this study was largely categorical and this could have contributed to the sensitivity of the results due to that categorical data generally has low sensitivity as opposed to numerical data. There are also few limitations to the chi-square test which is the main statistical test applied in this study. Firstly, the chi-square test is very sensitive to sample size. With a large enough sample like for this current study, even trivial relationships can appear to be statistically significant [29]. When using the chi-square test, one should keep in mind that “statistically significant” doesn’t necessarily mean “meaningful” [29]. Secondly, it must be noted that the chi-square can only tell us whether two variables are related to one another, it does not necessarily imply that one variable has any causal effect on the other [29]. In order to establish causality, a more detailed analysis would be required. Even though these are the biases we have to be aware of, they have been accounted for through random sampling, and appropriate analysis methods. Moreover, adequate data was not available for those whose IMCI training was unknown (e.g., year of training and other characteristics) and that is a study limitation by itself. Furthermore, the study questionnaire was not specific to whether a participant was trained in IMCI at school or on the job or not trained at all, something that would have made stratification much clearer.

## Conclusion

Overall, the knowledge levels of IMCI trained HCWs was not significantly different from those not IMCI trained, translating to that IMCI training does not have an effect on knowledge levels of HCWs in management of childhood illness. This, however, should be interpreted with caution since it could be likely due to that the number of those IMCI trained was less than half of all the participants, and other limitations of the parent survey, i.e., the survey contained questions on knowledge dimension only instead of on KAP for a more elaborate analysis and the data was largely categorical. The findings of the study call for strengthened advocacy on the IMCI training program and proper monitoring of outcomes. This could be done through selected indicators by MoH and through KAP or longitudinal studies concurrently with IMCI surveys. This will eventually improve healthcare service delivery in Botswana.

## Supporting information

S1 File Interview of healthcare workers questionnaire. (PDF)

S2 File Data spreadsheet. (XLSX)

S3 File Additional tables & graphical information. (PDF)

## References

[1] Mupara LU, Lubbe JC. Implementation of the Integrated Management of Childhood Illnesses strategy: challenges and recommendations in Botswana. Glob Health Action. 2016;9(1):29417. doi: 10.3402/gha.v9.29417 26899774 PMC4761685

[2] Gera T, Shah D, Garner P, Richardson M, Sachdev HS. Integrated management of childhood illness (IMCI) strategy for children under five. Cochrane Database Syst Rev. 2016;2016(6):CD010123. doi: 10.1002/14651858.CD010123.pub2 27378094 PMC4943011

[3] Kilov K, Hildenwall H, Dube A, Zadutsa B, Banda L, Langton J, et al. Integrated management of childhood illnesses (IMCI): a mixed-methods study on implementation, knowledge and resource availability in Malawi. BMJ Paediatr Open. 2021;5(1):e001044. doi: 10.1136/bmjpo-2021-001044 34013071 PMC8098945

[4] World Health Organization. Integrated management of childhood illness: a WHO/UNICEF initiative- Bulletin of the World Health Organization. 1997;75(1). Available from: https://iris.who.int/handle/10665/42045

[5] Gove S. Integrated management of childhood illness by outpatient health workers: technical basis and overview. The WHO Working Group on Guidelines for Integrated Management of the Sick Child. Bull World Health Organ. 1997;75(1):7–24. 9529714 PMC2486995

[6] Lambrechts T, Bryce J, Orinda V. Integrated management of childhood illness: a summary of first experiences. Bull World Health Organ. 1999;77(7):582–94. 10444882 PMC2557705

[7] Muhe LM, Iriya N, Bundala F, Azayo M, Bakari MJ, Hussein A, et al. Evaluation of distance learning IMCI training program: the case of Tanzania. BMC Health Serv Res. 2018; 18(1):547. doi: 10.1186/s12913-018-3336-y 30001709 PMC6044076

[8] Isangula K, Ngadaya E, Manu A, Mmweteni M, Philbert D, Burengelo D, et al. Implementation of distance learning IMCI training in rural districts of Tanzania. BMC Health Serv Res. 2023;23(1):56. doi: 10.1186/s12913-023-09061-y 36658537 PMC9854197

[9] Rakha MA, Abdelmoneim A-NM, Farhoud S, Pièche S, Cousens S, Daelmans B, et al. Does implementation of the IMCI strategy have an impact on child mortality? A retrospective analysis of routine data from Egypt. BMJ Open. 2013;3(1):e001852. doi: 10.1136/bmjopen-2012-001852 23355663 PMC3563136

[10] Schellenberg JRA, Adam T, Mshinda H, Masanja H, Kabadi G, Mukasa O, et al. Effectiveness and cost of facility-based integrated management of childhood illness (IMCI) in Tanzania. The Lancet. 2004;364(9445):1583–94. doi: 10.1016/s0140-6736(04)17311-x 15519628

[11] World Health Organization. Child Health and Development: Integrated management of childhood illness. WHO. 2024 [cited 2024 June 1]. Available from: https://www.who.int/teams/maternal-newborn-child-adolescent-health-and-ageing/child-health/integrated-management-of-childhood-illness

[12] Meaney PA, Joyce CL, Setlhare S, Smith HE, Mensinger JL, Zhang B, et al. Knowledge acquisition and retention following Saving Children’s Lives course for healthcare providers in Botswana: a longitudinal cohort study. BMJ Open. 2019;9(8):e029575. doi: 10.1136/bmjopen-2019-029575 31420392 PMC6701641

[13] Nkomazana O, Phaladze N, Peersman W, Willcox M, Mash R. Human resources for health in Botswana: the results of in-country database and reports analysis. Afr J Prim Health Care Fam Med. 2014;6(1):1–8. doi: 10.4102/phcfm.v6i1.716 26245420 PMC4564932

[14] Goga AE, Muhe LM. Global challenges with scale-up of the integrated management of childhood illness strategy: results of a multi-country survey. BMC Public Health. 2011;11(1):503. doi: 10.1186/1471-2458-11-503 21708029 PMC3155839

[15] Goga AE, Muhe LM, Forsyth K, Chopra M, Aboubaker S, Martines J, et al. Results of a multi-country exploratory survey of approaches and methods for IMCI case management training. Health Res Policy Sys. 2009;7(1):18. doi: 10.1186/1478-4505-7-18 19615080 PMC2723104

[16] Ramji S. Integrated management of neonatal and childhood illness (IMNCI): implementation challenges in India. Indian Pediatr. 2006;43(12):1029–31. 17202598

[17] Titaley C, Jusril H, Ariawan I, Soeharno N, Setiawan T, Weber M. Challenges to the implementation of the integrated management of childhood illness (IMCI) at community health centres in West Java province, Indonesia. WHO South East Asia J Public Health. 2014;3(2):161–70. doi: 10.4103/2224-3151.206732 28607302

[18] World Health Organization. Department of child and adolescent health and development. health facility survey: tool to evaluate the quality of care delivered to sick children attending outpatient facilities. WHO; 2003.

[19] World Health Organization. Harmonized health facility assessment (HHFA): Core questions. WHO; 2021 [cited 2024 May 15]. Available from: https://www.who.int/publications/i/item/harmonized-health-facility-assessment-(hhfa

[20] United Nations Development Program. Sustainable development goals- SDG 3: Good Health and Well being. UNDP; 2023 [cited 2024 May 29]. Available from: https://www.undp.org/sustainable-development-goals/good-health

[21] Ministry of Health, Botswana. Integrated management of childhood illness- 2017 health facility survey report. In. Gaborone: Ministry of Health & Wellness; 2017:32.

[22] Ministry of Health, Botswsna. Botswana 2012 IMCI health facility survey report. In. Gaborone: Ministry of Health; 2012:40.

[23] Mupara LM. Challenges identified by experienced IMCI-1-trained registered nurses in implementing the integrated management of childhood illnesses (IMCI) strategy in Gaborone, Botswana. 2013.

[24] Al-Yahyahi M, Al Kiyumi M, Jaju S, Al Saadoon M. Perceptions of undergraduate medical students toward integrated management of childhood illness (IMCI) pre-service education at Sultan Qaboos University, Muscat. Cureus. 2023;15(10):e47260. doi: 10.7759/cureus.47260 38022356 PMC10655620

[25] Nkosi Z, Botshabelo R, Jorosi H, Makole N, Nkomo G, Ruele S. The implementation of the integrated management of childhood illnesses (IMCI) strategy guidelines in Botswana. Africa Journal of Nursing and Midwifery. 2012;14(2):90–103.

[26] Mayhew M, Ickx P, Newbrander W, Stanekzai H, Alawi SA. Long and short integrated management of childhood illness (IMCI) training courses in Afghanistan: a cross-sectional cohort comparison of post-course knowledge and performance. Int J Health Policy Manag. 2015;4(3):143–52. doi: 10.15171/ijhpm.2015.17 25774371 PMC4357981

[27] Ariff S, Sadiq K, Jiwani U, Ahmed K, Nuzhat K, Ahmed S, et al. Evaluation the effectiveness of abridged IMNCI (7-day) course v standard (11-day) course in Pakistan. Matern Child Health J. 2022;26(3):530–6. doi: 10.1007/s10995-021-03276-3 34669101 PMC8917018

[28] Sylvies F, Nyirenda L, Blair A, Baltzell K. The impact of pulse oximetry and integrated management of childhood illness (IMCI) training on antibiotic prescribing practices in rural Malawi: a mixed-methods study. PLoS One. 2020;15(11):e0242440. doi: 10.1371/journal.pone.0242440 33211744 PMC7676725

[29] The University of Utah, Department of Sociology. The chi square test of independence. Salt Lake City, Utah; 2024 [cited 2024 May 31]. Available from: https://soc.utah.edu/sociology3112/chi-square.php

