# Supplementary material for: The Impact of integrated management of childhood illness training on knowledge levels of healthcare workers in Botswana
Source: PLOS Glob Public Health. 2025 Feb 24;5(2):e0003899. doi: 10.1371/journal.pgph.0003899 (PMC11849844; doi:10.1371/journal.pgph.0003899)
Supplement: S1 File — (PDF) [file pgph.0003899.s001.pdf]

Form 5. Interview of healthcare workers

Participant ID ____ District: ____ Sex: ____

Age of participant: ________________

Health facility: ______________________ Facility type: **Hospital/clinic/Health Post**

Cadre (circle): **General nurse/ Family nurse practioner/Midwife/General doctor/ Specialist Doctor/**

Other____

Duration of employment (years)_____________ Trained in IMCI: (Yes/No)

Year of training: ____

Highest Qualifications: **Diploma, Degree, Masters or higher**

Duration of work at this facility (years): ______________

1.Do you consult sick children at the facility*? (*Yes/*No)*

If 'NO' go to question 4

2. Do you use the integrated management of childhood illness (IMCI) guidelines on your practice? **(Yes/No)**

3. How useful do you think the IMCI guidelines in the clinical management of sick children?
Very useful Useful Not very useful Not at all useful

4.According to the IMCI guidelines, what are the danger signs?

Danger signs

- Mentions the child not able to eat or drink: **(Yes / No)**
- Mentions the child vomits everything or unable to retain feeds: **(Yes/ No)**
- Mentions the child has convulsions: **(Yes/ No)**

4.What is the case definition of Acute Flaccid Paralysis?

- Mentions acute weakness: **(Yes/ No)**
- Mentions flaccid/floppy: **(Yes/ No)**
- Mentions weakness in any age group regardless of age: **(Yes/ No)**
- Mentions age under 15 years: **(Yes/ No)**

5.What is the case definition of measles?

- Mentions fever and rush: **(Yes/ No)**

6.What is the case definition of diarrhea?

- Mentions 3 or more loose stools in a day: **(Yes/ No)**

7.What are the signs of severe diarrhea?

- Lethargic or unconscious: **(Yes/ No)**
- Inability to drink or breast feed: **(Yes/ No)**
- Sunken eyes: **(Yes/ No)**
- The child vomits everything/ cannot retain feeds: **(Yes/ No)**
- Skin pinch goes back slowly: **(Yes/ No)**

8.What are the signs of severe measles?

- Clouding cornea: **(Yes/ No)**
- Deep or extensive mouth ulcers: **(Yes/ No)**

9.How do you assess the nutritional status of a child?

- Mentions mid upper arm circumference: **(Yes /No)**
- Mentions measuring the patient's weight: **(Yes /No)**
- Mentions measuring the patient's height: **(Yes /No)**
- Mentions measuring weight for length or weight for height: **(Yes/ No)**
- Mentions examination for edema: **(Yes/ No)**

Final remarks/Comments

____
____
____
____
____
____
