# Supplementary material for: The Impact of integrated management of childhood illness training on knowledge levels of healthcare workers in Botswana
Source: PLOS Glob Public Health. 2025 Feb 24;5(2):e0003899. doi: 10.1371/journal.pgph.0003899 (PMC11849844; doi:10.1371/journal.pgph.0003899)
Supplement: S3 File — (PDF) [file pgph.0003899.s003.pdf]

Supporting information: Additional Tables & Graphical Information

Table 5 below shows the performance of those who consult sick children only on IMCI knowledge questions. Most HCWs, 411 (66.8%) indicated that IMCI guidelines are very useful in the clinical management of sick children. Generally most HCWs were not able to adequately state all the three general danger signs according to IMCI guidelines. Majority, 572 (93.0%) were able to state the case definition of measles and similarly 567 (92.2%) were able to adequately state the case definition of diarrhoea. There was variability in getting the signs of severe diarrhoea right, for some majority got them right while for some majority did not get them. For stating the signs of severe measles 411 (66.8%) and 476 (77.4%) did not get it right that "clouding cornea" and "deep or extensive mouth ulcers" are signs of severe measles respectively. For assessment of nutritional status of a child: 403 (65.5%) mentioned mid upper arm circumference, 578 (94.0%) mentioned measuring the patient's weight, and 386 (62.8%) mentioned measuring the patient's height. On the other hand, 320 (52.0%) did not mention measuring weight for length or weight for height and 418 (68.0%) did not mention examination for edema as part of assessment for nutritional status of a child.

Table 5: Performance of healthcare workers who consult sick children on knowledge questions (questions 3-9 of the questionnaire)

**Knowledge Questions**	**Very useful (%)**	**Useful (%)**	**Not very useful (%)**	**Not useful at all (%)**	**No answer (%)**
**3. How useful do you think the IMCI guidelines are in the clinical management of sick children?**	411 (66.8)	158 (25.7)	6 (1.0)	3 (0.5)	37 (6.0)

		**Yes (%)**	**No (%)**
**4a. According to the IMCI guidelines, what are the general danger signs?**			
	Mentions the child not able to eat or drink:	297 (48.3)	318 (51.7)

	Mentions the child vomits everything or unable to retain feeds:	299 (48.6)	316 (51.4)
	Mentions the child has convulsions:	338 (55.0)	277 (45.0)
**4b. What is the case definition of Acute Flaccid Paralysis?**			
	Mentions acute weakness:	438 (71.2)	177 (28.8)
	Mentions flaccid/floppy:	275 (44.7)	340 (55.3)
	Mentions weakness in any age group regardless of age:	86 (14.5)	526 (85.5)
	Mentions age under 15 years:	131 (21.3)	484 (78.7)
**5.What is the case definition of measles?**			
	Mentions fever and rash:	572 (93.0)	43 (7.0)
**6.What is the case definition of diarrhoea?**			
	Mentions 3 or more loose stools in a day:	567 (92.2)	48 (7.8)
**7.What are the signs of severe diarrhoea?**			
	Lethargic or unconscious:	448 (72.9)	167 (27.2)
	Inability to drink or breast feed:	239 (38.9)	376 (61.1)
	Sunken eyes:	559 (90.9)	56 (9.1)
	The child vomits everything/ cannot retain feeds:	161 (26.2)	454 (73.8)
	Skin pinch goes back slowly:	562 (91.4)	53 (8.6)

Table 4: (continuation)

		**Yes (%)**	**No (%)**
**8.What are the signs of severe measles?**			
	Clouding cornea:	204 (33.1)	411 (66.8)
	Deep or extensive mouth ulcers:	139 (22.6)	476 (77.4)
**9.How do you assess the nutritional status of a child?**			
	Mentions mid upper arm circumference:	403 (65.5)	212 (34.5)
	Mentions measuring the patient's weight:	578 (94.0)	37 (6.0)
	Mentions measuring the patient's height:	386 (62.8)	229 (37.2)
	Mentions measuring weight for length or weight for height:	295 (48.0)	320 (52.0)
	Mentions examination for edema:	197 (32.0)	418 (68.0)

The healthcare workers performance on the questions on diagnosis and assessment for common childhood illnesses is summarised by figure 1 below. The total knowledge scores of each individual were computed by adding their individual scores for each question. The maximum knowledge score was 21. The knowledge of individual healthcare workers were categorised according to Bloom's cutoffs, where 80-100% represents good performance, 60-79% represent moderate performance, and < 60% represents poor performance. This approach is consistent with previous studies [24–26]. Only 12.5% and 20.3 % of individuals achieved good and moderate performance respectively according to these cutoffs. The majority of healthcare workers (67.2%) had poor performance. There was no significant difference in performance according to IMCI training. Only 13.9 % of IMCI trained individuals achieved good performance compared to 10.5% of individuals who are not trained on IMCI. Similarly, 64.1% of IMCI trained individuals had poor performance compared to 71.7% of individuals not trained on IMCI.

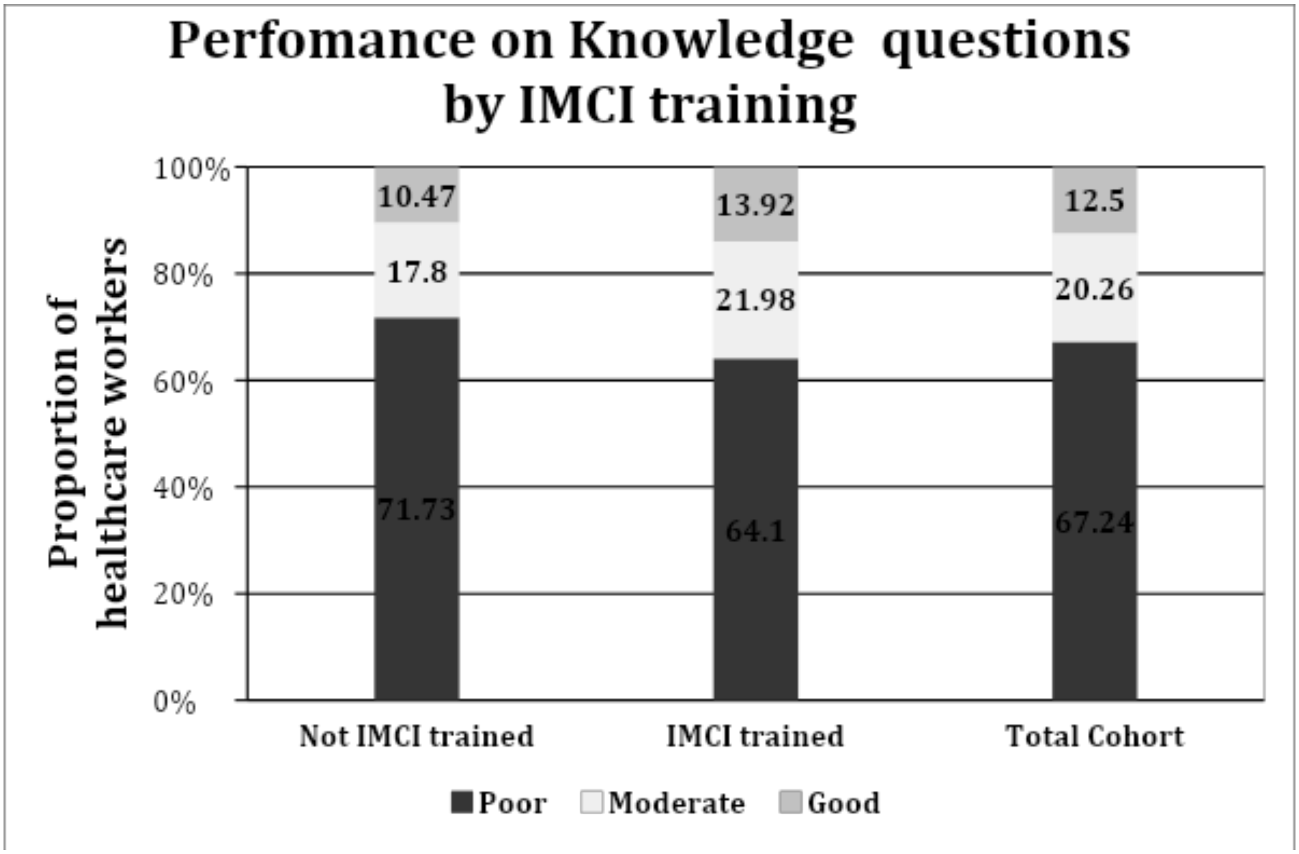

Figure 1. Performance on Knowledge questions by IMCI questions

Figure 2 below shows HCWs who were not able to mention all of the 3 selected general danger signs. Among the IMCI trained HCWs, only 10.5% of HCWs were able to mention all the 3 general danger signs. Similarly, only 6.8% of HCWs not trained on IMCI were able to mention the 3 general danger signs. Overall, 9.0% of healthcare workers interviewed were able to mention the 3 general danger signs.

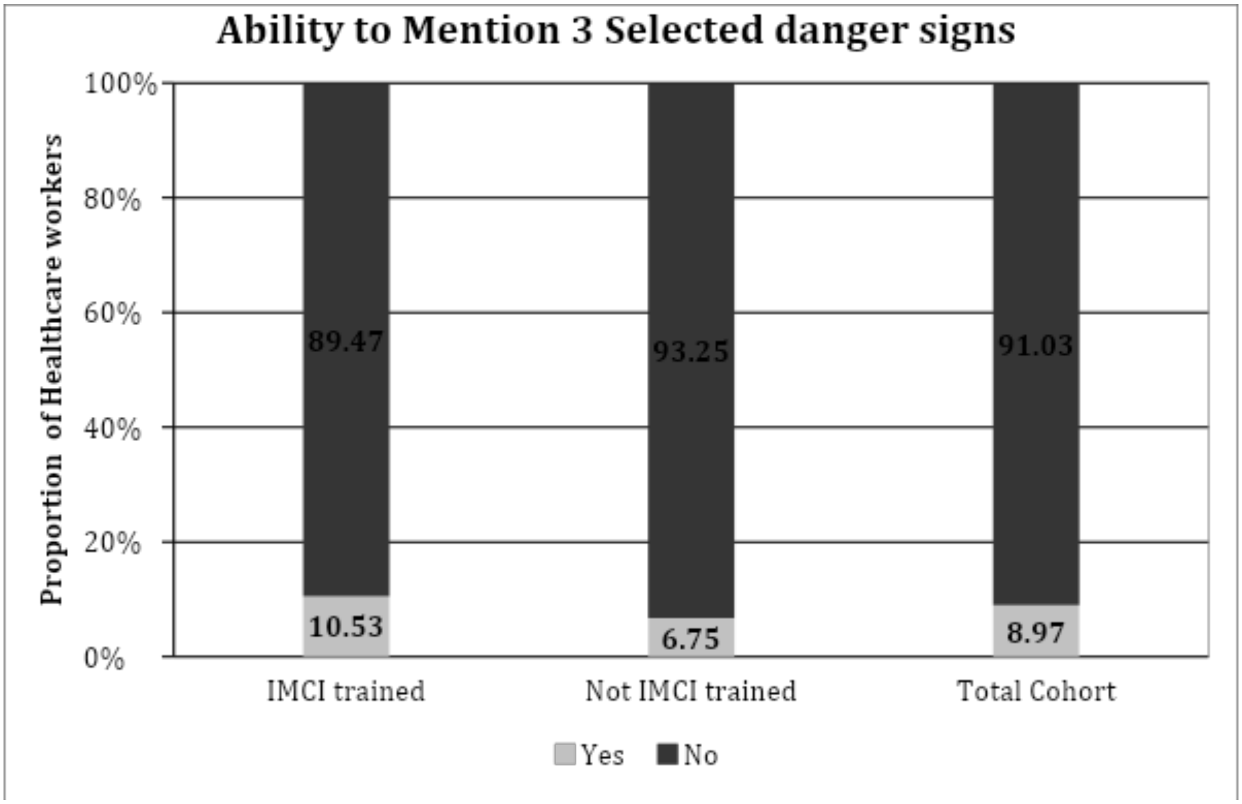

Figure 2. Performance on knowledge of general danger signs

The IMCI training status of different cadres is displayed by figure 3 below. Eight (8) of the 26 doctors were trained in IMCI while 18 of them were not IMCI trained. The most IMCI trained cadre was general nurse at 239 while 159 of them were not IMCI trained. Twenty-six (26) post basic nurses were IMCI trained while 15 were not. For other cadres only 190 were trained while 287 were not trained.

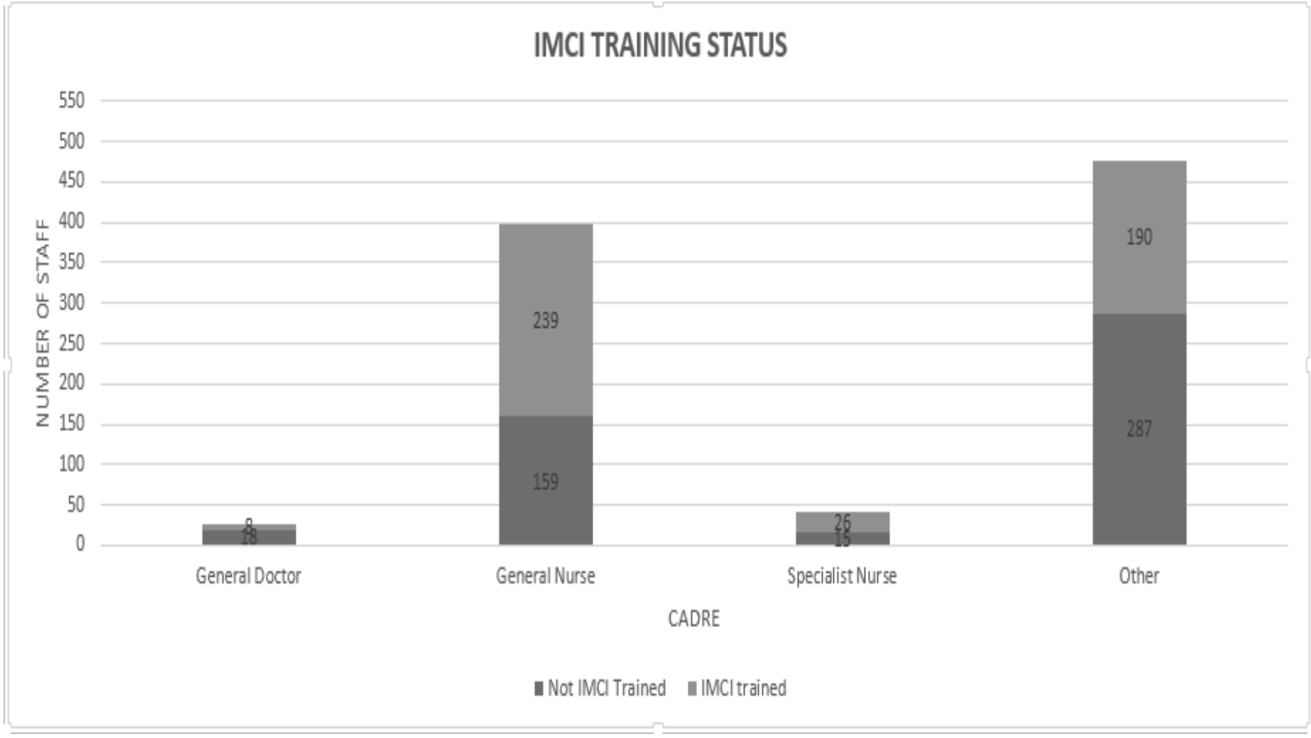

Figure 3. IMCI Training Status
